# Stat5 deficiency decreases transcriptional heterogeneity and supports emergence of hematopoietic sub-populations

**DOI:** 10.18632/oncotarget.15236

**Published:** 2017-02-09

**Authors:** Zhengqi Wang, Kevin D. Bunting

**Affiliations:** ^1^ Department of Pediatrics, Aflac Cancer and Blood Disorders Center of Children's Healthcare of Atlanta and Emory University School of Medicine, Atlanta, GA, USA

**Keywords:** hematopoiesis, transcription factor, single-cell gene expression analysis, transcriptional heterogeneity, leukemogenesis

## Abstract

Aging is associated with significant changes in hematopoiesis, including clonal dominance, anemia, myeloid malignancies, and reduced activation of signal transducer and activator of transcription 5 (Stat5). In previous studies, Stat5 deletion surprisingly amplified FLT3/ITD^+^ myeloid expansion or Myc-driven lymphoid expansion. Here we show that Stat5 deficiency has a strong impact upon transcriptional heterogeneity in single sorted c-Kit^+^Lin^−^Sca-1^+^ (KLS) cells or CD150^+^CD48^−^ KLS long-term repopulating hematopoietic stem cells (LT-HSC). Single cell polymerase chain reaction (PCR) was performed on selected regulators of multi-lineage hematopoiesis. At least two dominant sub-populations were identified by increased expression of cell cycle regulatory and leukemia-associated genes. Furthermore, in the top expressing quartile of cells, the majority of genes were proportionally overrepresented. In wild-type KLS cells, Stat5 mRNA levels were also strongly correlated with several genes. Since heterogeneity decreases with age or inflammatory or oncogenic stress, these results provide a potential mechanistic linkage to Stat5 expression.

## INTRODUCTION

Stat5 is critical for normal development of lymphocytes and erythrocytes and for hematopoietic stem cell (HSC) repopulating activity, quiescence, and self-renewal [1]. While gene regulation by Stat5 has been defined in cell culture systems and bulk tissue populations, transcriptional regulation is known to be cell type and context dependent. Since heterogeneity decreases with age [2, 3] and is also associated with reduced levels of phosphorylated Stat5 [4, 5], we sought to characterize the biological effects of Stat5 deletion on transcriptional heterogeneity in early hematopoiesis. Therefore, we utilized Stat5 deficient mice to test the biological impact of Stat5 deletion on transcriptional heterogeneity in single sorted c-Kit^+^Lin^−^Sca-1^+^ (KLS) cells or CD150^+^CD48^−^ KLS long-term repopulating hematopoietic stem cells (LT-HSC). Single cell polymerase chain reaction (PCR) was performed using the Fluidigm Biomark system on a selected set of genes that are critical regulators of multi-lineage hematopoiesis. We observed expression changes consistent with Stat5-independent HSC lineage-commitment. The results may give new molecular insight into age-related clonal hematopoiesis and pre-leukemic stem cells [6–10] in humans.

## RESULTS AND DISCUSSION

Study of hematopoiesis at the single cell level provides key insights into multilineage priming of HSCs and changes at the sub-population level in KLS cells. Here we describe the impact of loss of Stat5 [11] on gene expression and demonstrate a novel role in suppression of sub-populations with elevated expression of cell cycle regulatory and leukemia-associated genes. Wild-type and Stat5ab^null/null^ fetal liver transplanted chimeric mice were generated by transplanting E14.5 fetal livers into lethally irradiated recipients and analyzing at least 3 months later. Single LT-HSC or KLS cells were sorted directly into 96-well PCR plates and real-time single cell PCR analysis was carried out using the Fluidigm BioMark 96.96 array system according to the Fluidigm protocol. Two separate analyses from independent sorting and each with two technical replicates were normalized based on the mean of average of Ct of whole array.

**Figure 1:**
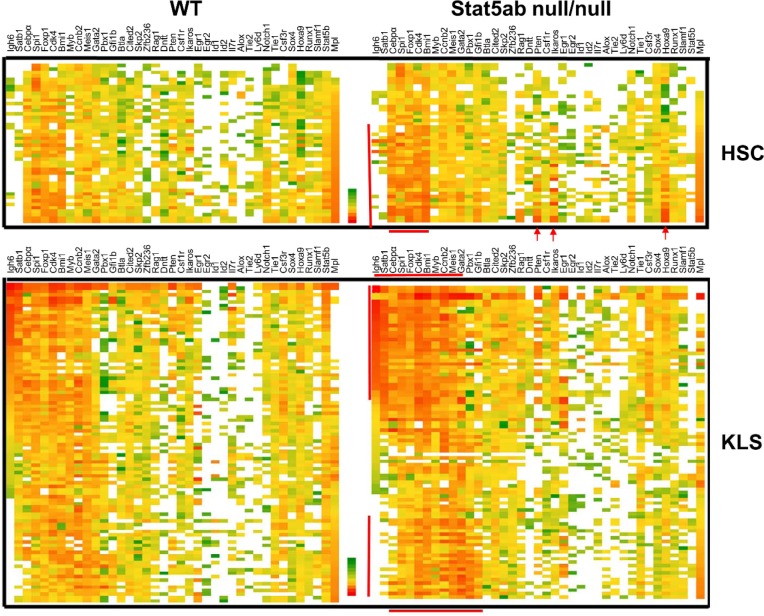
Transcriptional heterogeneity in Stat5 deficient LT-HSC and KLS cells analyzed by single cell PCR Single cell PCR was performed twice using a Fluidigm 96.96 dynamic array with sorted LT-HSC or KLS cells from either wild-type or Stat5ab^null/null^ fetal liver transplanted chimeric mice. The top heat map depicts all the single cells (23 cells each) and 39 genes analyzed with sorted Stat5 wild type and knockout LT-HSC (CD150^+^CD48^−^KLS) cells. Heat maps were created from Excel Pivot tables with conditional formatting (Excel 2010). Red/Orange represents a high level of expression (lower Ct) and green represents a lower level of expression (Higher Ct). White represents failure to detect. The bottom heat map shows KLS cells (46 cells for WT and 45 cells for Stat5ab^null/null^) that were from independent flow cytometric sorting from two different batches of fetal liver chimeric mice. Bone marrow cells were pooled from 2-3 chimeric mice. Any primer sets that failed to detect signal from 10 cells of the positive control were removed from analysis. The mean average of Ct values from the whole array was normalized to the same between arrays. Sub-population cells are bracketed by red lines or arrows.

**Figure 2:**
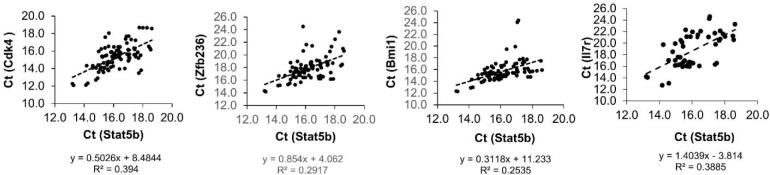
The correlation coefficient between Stat5b and the rest of the genes Correlation was calculated using single cell PCR data from WT KLS cells. Pairs of genes with strong correlations were plotted with a linear regression trend line. The linear regression equation and R-squared value is displayed in each plot. Cdk4, Zfb236, Bmi1, and Il7r had the strongest associations with Stat5b.

To compare Stat5-mediated gene expression between wild type (WT) and knockout (KO) LT-HSC and KLS cells, genes were selected from the literature as a sample of key regulators with documented roles in self-renewal, quiescence, and lineage commitment. We performed single cell PCR on 46 single WT and 45 Stat5 KO KLS cells and 23 single WT and 23 Stat5 KO LT-HSCs using two Fluidigm BioMark 96.96 arrays with normalization for the average Ct. The full data sets of all primers tested on KLS cells are shown in Supplementary Figure 1 in a violin plot which is similar to a box plot with the addition of showing probability density of the data at different values. As an important control, expression of Stat5 was dramatically lost in the knockout cells relative to the wild-type. Surprisingly, there were more cells with deficiency of Stat5 having relatively higher levels of gene expression (lower Ct) than that of the wild type control. To better visualize overall gene expression changes in sub-populations of cells, the data is shown as a heat map (Figure 1) with the same single cell represented across each row for either LT-HSC or KLS cells. As expected, Mpl expression was inversely related to the expression of lymphoid lineage genes such as Igh6 and Satb1 [12]. Two heavily overlapping clusters of genes including lymphoid-myeloid multilineage (Igh6, Satb1, Cebpα, Spi1, Foxp1, Cdk4, Bmi1, Myb, Ccnb2, Meis1, Gata2) or myeloid multilineage (Spi1, Foxp1, Cdk4, Bmi1, Myb, Ccnb2, Meis1, Gata2, Pbx1, Gfi1b) were prominently increased in expression in Stat5 KO KLS cells (bracketed by red lines). In LT-HSC, there was increased priming of a myeloid multilineage sub-population with relatively elevated expression of Cebpα, Spi1, Foxp1, Cdk4, Bmi1, Pten, Ikaros, and Hoxa9. Notably, besides Stat5b and Mpl, very few genes had significantly decreased expression in Stat5 KO KLS cells. Mpl was reduced more in long-term repopulating HSCs (LT-HSCs). Interestingly, in KLS cells wild-type Stat5b mRNA also correlated strongly with several genes (Figure 2) including Cdk4 and Bmi1, consistent with dosage-dependent Stat5 effects.

**Figure 3:**
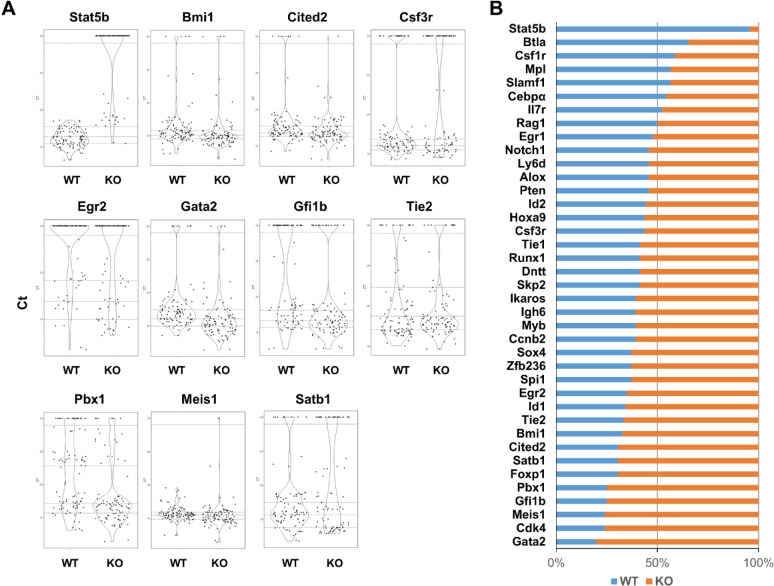
The majority of the key regulatory genes within the highest expressing KLS cells are overrepresented in the absence of Stat5 **A**. KLS were re-analyzed for genes with significant changes in expression based on the Mantel-Haenszel Chi-squared test with P value less than 0.05 (for details see Materials and Methods), and plotted on violin plots with R software. The y-axis for each violin plot varied depending on the relative expression level and the distribution between the highest and lowest expressing cells. The dotted lines separate data into five categories: The Ct with arbitrarily set value at 30 as a separate category with no or extremely low level expression. The remaining Ct values from each gene are categorized into 4 quartiles. **B**. The rank test was applied to each gene expression in Stat5 WT and KO KLS cells. The Ct value from each gene in Stat5 WT and KO single KLS cell was ranked from low to high. The highest expression (the top 25% of cells with low Ct value) for each gene was further checked for the distribution between WT and KO cells. Blue represents the percentage of WT KLS cells with the highest gene expression and orange represents the percentage of KO KLS cells with the highest gene expression.

We also divided gene expression into groups. The first group was defined as those with undetectable expression and the other groups with detectable levels of expression were sub-divided into four quartiles based on Ct value. The difference of gene expression between Stat5 WT and KO was checked with Mantel-Haenszel Chi-Square test or Fisher exact test in cases of extremely low events in subpopulation. There were 11/39 genes with significantly changed overall gene expression in Stat5 KO KLS cells compared to WT KLS cells (*p* < 0.05) (Figure 3A). Among them, Stat5b, Gfi1b, Pbx1, Gata2 and Cited2 had the most significant changes (*p* < 0.001). Additionally, Bmi1, Csf3r, and Tie2 (*p* < 0.01) as well as Egr2, Meis1 and Satb1 (*p* < 0.05) were significantly changed. Furthermore, when the stringency for gene expression changes was focused on the top 25% expressing cells in the combined data from both WT and Stat5 KO, shifts in gene representation were most clearly observed for the majority of Stat5 KO KLS cells (Figure 3B).

An important observation of this study is a core signature of genes that were increased in expression in a substantial proportion of single cells lacking Stat5. There is abundant evidence defining cell-type specific Stat5 target genes. However, less is known about how Stat5 modulates gene expression. Although we did not define whether gene expression changes are direct or indirect, the emergence of sub-populations is strongly suggestive of a selective growth advantage. It is becoming clear that mixed lineage states exist, even transiently, during hematopoiesis as revealed at the single cell level [13]. Additionally, combinatorial transcription factor interactions are major drivers of mutllineage hematopoietic development through cooperative transcriptional control [14] which could promote transcriptional heterogeneity of myeloid progenitors [15]. Since two main clusters of genes were increased in two sub-populations, increased gene expression in Stat5 KO KLS cells is consistent with this clonal understanding. Genes were associated with cell cycle regulation (Cdk4 and Cyclin B2), preserving lymphoid or myeloid potential (Satb1, Pbx1 and Gfi1b) or induction of leukemia stem cell self-renewal (Bmi1 and Meis1) consistent with prior studies [16, 17]. Aberrant expression of Bmi1, Meis1/Pbx1, and Cdk4 have been implicated in leukemogenesis [18–20].

Overall, we conclude that Stat5 has a role in myeloid multilineage priming in murine LT-HSCs and regulates myeloid and lymphoid-myeloid multilineage progenitor emergence from HSCs. This is the first demonstration of a linkage between decreased Stat5 activation in early hematopoiesis and emergence of lymphoid-myeloid multilineage sub-populations. Interestingly, Stat5 deletion in this mouse model promoted faster development of Myc-driven B-cell acute lymphoblastic leukemia [21] and promoted granulocyte-monocyte progenitor expansion and myeloproliferative neoplasm phenotypes such as splenomegaly in a Flt3ITD/+ transgenic mouse model [22]. These prior studies provide key functional support that the transcriptional changes documented here are of significance in the setting of oncogene-driven hematopoiesis. Considering that hematopoietic differentiation is required for initiation of AML [23], further studies under different stress conditions, including inflamm-aging [24] may uncover additional insight into the role of Stat5.

Notably, we found that at least two unique sub-populations emerged, characterized by increased expression of cell cycle regulatory and leukemia-associated genes. Furthermore, in the top expressing quartile of cells, the majority of genes were proportionally overrepresented in the absence of Stat5 with the biggest shifts observed for Gata2 and Cdk4 expressing cells. In wild-type KLS cells, Stat5 mRNA levels were also strongly correlated with several genes, including Cdk4 suggesting a dual regulatory relationship. It is interesting that the changes in KLS gene expression involves medium/high expressing genes that increase in expression. It is possible that such changes reflect a normal role for Stat5 in moderating gene expression at the clonal level. Therefore, Stat5 plays an important role in maintenance of normal transcriptional heterogeneity of hematopoietic progenitors where it has an unexpectedly prominent function to modulate clonal balance. The findings promote better understanding of Stat5 function during aging and leukemogenesis. Since Stat5 is also a potential direct and/or indirect molecular target for hematologic disease, the changes in gene expression described here may need to be considered in pre-leukemic hematopoietic progenitors, during therapy, or during clonal changes associated with relapse. Interestingly, Porter et al. [22] recently demonstrated that competence for Flt3ITD^+^/Stat5 target gene expression was found in adult (> 2 weeks) but not fetal or neonatal mice. Therefore, childhood AML is afforded a relative protection against leukemogenesis and concerns about Stat5 inhibition as therapy may be more relevant for the adult population.

## MATERIALS AND METHODS

### Fetal liver stem cell transplantation

Wild-type and Stat5ab^null/null^ fetal liver transplanted chimeric mice were generated by transplanting E14.5 fetal livers (obtained from timed mating of Stat5ab^+/null^ X Stat5ab^+/null^ mice) with 1 donor fetal liver into 5 lethally irradiated recipients. 3 months after transplantation, donor derived KLS and LT-HSC (CD150+CD48-KLS) were sorted by flow cytometry.

### Real-time single cell PCR analysis

Single cells were sorted directly into 96-well PCR plates containing 5 μL of CellsDirect 2x reaction mix with SUPERase-In RNase Inhibitor (Ambion). RT and target specific amplifications were carried out at 50°C, 15 min; then 95°C 2 min; 18 cycles of 95°C for 15 seconds and 60°C for 4 min. Then the samples were treated with Exonuclease I to eliminate the carryover of unincorporated primers and further diluted 1:5 in TE buffer and analyzed on BioMark 96.96 array.

### Single cell PCR data analysis

Two technical replicates from the same sort were included for each cell population. For 96.96 array data two separate analyses from independent sorting and each with two technical replicates was normalized based on the mean of average of Ct of whole array. Heat maps were created from Excel Pivot tables with conditional formatting (Excel 2010). Violin plots were generated with R software (version 3.3.0) for Windows using ggplot2 with jitter function. The overall PCR failure rate was relatively low (only 5% of primer sets tested). All successful amplification data has been included.

### Statistical analysis

For single cell PCR, Ct from all cells from each primer set was sub-divided into five categories. The cells with undetectable gene expression (an arbitrary Ct value of 30 was assigned) were set as one category. For the remaining cells, the Ct for each primer set was categorized into 4 quartiles. Mantel-Haenszel Chi-squared analysis was used to test the association between the groups (groups were the wild type and knockout) and the quartiles. The analysis was conducted using SAS 9.4 (SAS Institute, Cary, North Carolina).

## SUPPLEMENTARY MATERIALS FIGURE


