# Supplementary material for: Stat5 deficiency decreases transcriptional heterogeneity and supports emergence of hematopoietic sub-populations
Source: Oncotarget. 2017 Feb 9;8(14):22477–82. doi: 10.18632/oncotarget.15236 (PMC5410237; doi:10.18632/oncotarget.15236)
Supplement: Supplementary file 1 [file oncotarget-08-22477-s001.pdf]

Stat5 deficiency decreases transcriptional heterogeneity and supports emergence of hematopoietic sub-populations

Supplementary Material

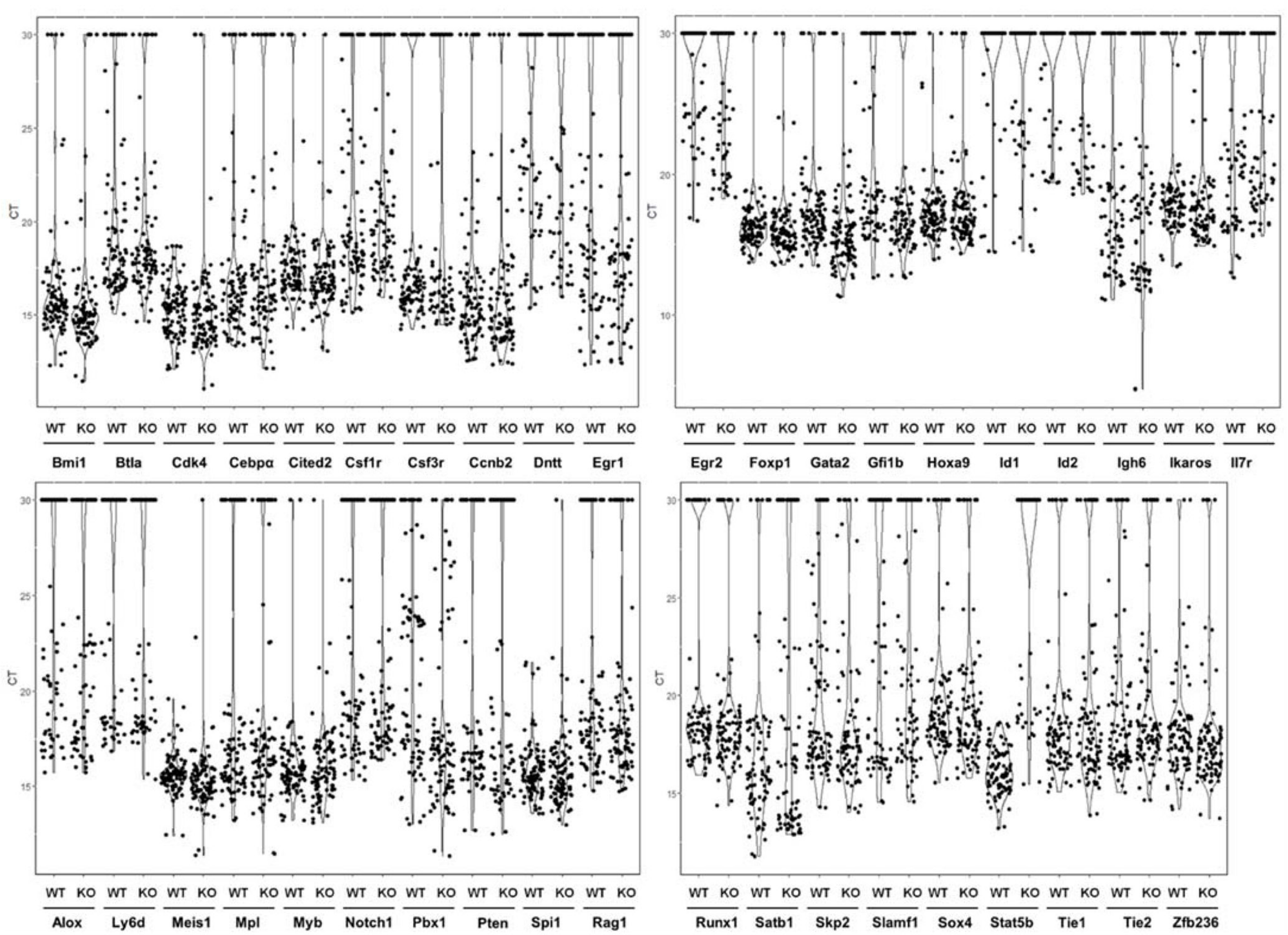

Supplemental Figure 1: Violin plots of data from two Fluidigm 96.96 dynamic arrays comparing wild-type and STAT5ab null/null sorted single KLS cells. Single cell PCR was performed twice using a Fluidigm 96.96 dynamic array with sorted KLS cells from either wild-type or Stat5ab$^{null/null}$ fetal liver transplanted chimeric mice three months after transplantation. KLS cells for each array were from independent flow cytometric sorting from two different batches of fetal liver chimeric mice. Bone marrow cells were pooled from 2-3 chimeric mice. Any primer sets that failed to detect signal from 10 cells of the positive control were removed from analysis. The mean average of Ct values from the whole array after removing failed primer sets was normalized to be the same between arrays. An arbitrary Ct value of 30, which is higher than any other Ct value obtained from the dynamic array, was assigned to any cell without detectable signal. Violin plots were generated with R software for Windows (version 3.3) using ggplot2 with jitter function (www.r-project.org).
